# Mapping food access: how neighborhood deprivation shapes healthy food availability in the United Kingdom

**DOI:** 10.1186/s12889-026-26664-2

**Published:** 2026-02-23

**Authors:** May A. Beydoun, Michael F. Georgescu, Sri Banerjee, Hind A. Beydoun, Nicole Noren Hooten, Jagdish Khubchandani, Ana I. Maldonado, Jack Tsai, Jordan Weiss, Michele K. Evans, Alan B. Zonderman

**Affiliations:** 1 Laboratory of Epidemiology and Population Sciences, National Institute on Aging, NIA/NIH/IRP, 251 Bayview Blvd., Suite 100, 04B118, Baltimore, MD 21224 USA; 2 Public Health Program, Walden University, Minneapolis, MN 55415 USA; 3 VA National Center On Homelessness Among Veterans, U.S. Department of Veterans Affairs, Washington, DC 20420 USA; 4 Department of Management, School of Public Health, and Community Health, University of Texas Health Science Center at Houston, PolicyHouston, TX 77030 USA; 5 College of Health, Education and Social Transformation, New Mexico State University, Las Cruces, NM USA; 6 U.S. Department of Veterans Affairs, Palo Alto, CA 94304 USA; 7 Optimal Aging Institute, New York University Grossman School of Medicine, New York, NY 10016 USA

**Keywords:** Food desert, Area deprivation, Geospatial analysis, EFDI, IMD, United Kingdom, Public health nutrition

## Abstract

**Background:**

Limited access to affordable, nutritious food is a key structural determinant of health inequality. In the United Kingdom, food desert risk is often assumed to mirror socioeconomic deprivation, yet their alignment across small-area geographies remains unclear.

**Objectives:**

To characterize the spatial distribution of food desert risk across England, Wales, and Scotland; quantify its association and concordance with area-level deprivation; and identify deprivation domains most strongly linked to food desert risk.

**Methods:**

We integrated the E-Food Desert Index (EFDI) with national Indices of Multiple Deprivation (IMD) across 41,729 Lower Layer Super Output Areas and Data Zones using 2011 Census boundaries. Standardized scores and quartiles were analyzed using spatial clustering, correlation, multinomial regression, ordinary least squares, adaptive LASSO with interaction terms, and mixed-effects and spatial sensitivity analyses.

**Results:**

Food desert risk and deprivation were moderately correlated (*r* = 0.40, *p* < 0.001) but showed substantial spatial discordance. Nearly half (48.2%) of the most deprived areas were also in the highest food desert quartile, while about one-third showed discordant classifications. Deprivation showed a strong graded association with food desert risk (RRR per IMD quartile = 3.07; 95% CI: 2.98–3.16). Employment deprivation was the strongest predictor across nations, followed by education, housing/access to services, and living environment. Antagonistic employment–income interactions were observed. Rural areas with low deprivation but high food desert risk were especially evident in Scotland.

**Conclusions:**

Food desert risk and deprivation represent overlapping but distinct dimensions of structural disadvantage. Reliance on deprivation indices alone may miss communities with substantial food access barriers, particularly in rural settings. Integrating food environment metrics alongside deprivation measures may improve place-based policy targeting.

**Supplementary Information:**

The online version contains supplementary material available at 10.1186/s12889-026-26664-2.

## Highlights

- Food desert risk and area deprivation were moderately correlated across 41,729 UK areas.
- Nearly half of the most deprived areas also faced the highest food desert risk.
- Employment deprivation was the strongest predictor of poor food access.
- EFDI and IMD capture overlapping but distinct structural determinants of health.
- Combining food-environment and deprivation metrics can better target underserved areas.

**Supplementary Information:**

The online version contains supplementary material available at 10.1186/s12889-026-26664-2.

## Introduction

Access to nutrient-rich and affordable food is a key determinant of population health [1]. In many United Kingdom (UK) regions, topographical and socioeconomic barriers impede individuals' ability to maintain a healthy diet [2–7]. Food deserts are areas with limited access to supermarkets or major grocery shops offering fresh produce and other nutritious options [2–7]. Nevertheless, these areas are typically marked by a greater concentration of fast food establishments and convenience stores, leading to suboptimal nutritional quality and an increased risk of chronic conditions, including obesity, diabetes, and cardiovascular disease [2–7].

Food deserts are closely tied to broader socioeconomic inequality [2–20]. Area deprivation, including income, employment, education, housing, and service access, is a well-established factor in health disparities in the UK and worldwide [2–20]. Disadvantaged areas are more likely to lack quality food retailers and infrastructure that supports healthy eating, such as public transit and walkable neighborhoods, and more recently access to e-commerce [5–7, 11, 12, 21–24]. Consequently, residents of these areas face both geographic and economic barriers to accessing nutritious food [5–7, 11, 12, 21–24]. Despite a growing literature on neighborhood food environments, several important gaps remain. Much of the existing UK research has examined food access, food deserts, or food swamps in isolation, often focusing on single cities, regions, or nations, or relying on proxy measures such as supermarket density or proximity alone. Fewer studies have explicitly linked multidimensional measures of food access disadvantage to validated, nationally standardized indices of socioeconomic deprivation at small-area resolution.

As highlighted in prior reviews (e.g., [12]), there is a need for geographically harmonized analyses that integrate food environment metrics with deprivation frameworks to better capture the structural nature of food access inequities and to inform policy-relevant targeting at scale. Moreover, while deprivation is frequently assumed to subsume food access disadvantage, empirical evidence quantifying their concordance and divergence across diverse geographic contexts remains limited.

The UK provides a uniquely informative setting in which to address these gaps. England, Wales, and Scotland share broadly similar welfare systems and public health infrastructures, yet differ meaningfully in settlement patterns, rurality, transport networks, retail landscapes, and the construction of their respective Indices of Multiple Deprivation. These cross-national contrasts within Great Britain offer an opportunity to examine whether relationships between food desert risk and deprivation are consistent or context dependent. Importantly, although the term “United Kingdom” is commonly used in public health discourse, the E-Food Desert Index is currently available only for Great Britain, necessitating a focused examination of England, Wales, and Scotland. Explicit consideration of these nations individually is therefore essential for both methodological transparency and interpretability. With interest in spatial food environments growing, more research is needed to link food desert prevalence at small-area levels to validated national measures of deprivation [4]. Understanding this relationship is essential for guiding policies designed to mitigate health disparities by enhancing food accessibility.

Accordingly, the objectives of this study were threefold: (1) to characterize the spatial distribution of food desert risk across small-area geographies in England, Wales, and Scotland; (2) to quantify the association and concordance between food desert risk and area-level deprivation using harmonized deprivation metrics; and (3) to identify which domains of deprivation are most strongly associated with food desert risk within and across nations. By integrating a multidimensional food access index with nationally recognized deprivation measures at high geographic resolution, this study aims to advance understanding of structural inequities in food environments across Great Britain and to inform more nuanced, place-based policy interventions.

## Methods

### Database

We used three open-access sources to examine the link between food desert status and area-level deprivation in England, Wales, and Scotland, now made available at:

https://data.hasp.ac.uk/browser/landing and https://data.geods.ac.uk/. The E-Food Desert Index (EFDI), developed by the Consumer Data Research Centre (CDRC), is a multidimensional index of food accessibility deprivation. The Index of Multiple Deprivation (IMD) captures area-level socioeconomic disadvantage across multiple domains and is generally thought of as a measure of relative deprivation in England and other parts of the UK that encompasses various facets of deprivation beyond simply low income. In addition to the proportion of the population experiencing income deprivation due to low income, including those out of work and those with low earnings, the IMD considers other domains of deprivation.

Both indices were harmonized using the 2011 Census Geography Boundaries (https://statistics.ukdataservice.ac.uk/dataset/2011-census-geography-boundaries-regions) [25] to enable cross-national comparisons and spatial integration. This harmonization allows consistent national coverage and supports robust spatial analyses across Great Britain. More specifically, we combined open data on food access and deprivation with 2011 Census boundaries for Lower Layer Super Output Areas (LSOAs) (England/Wales) and Data Zones (Scotland), enabling cross‑national harmonisation and mapping. LSOAs in England and Wales and Data Zones in Scotland are small-area statistical units designed to represent relatively homogeneous neighborhoods, each typically encompassing about 1,000–1,500 residents, and are widely used in UK public health research because they provide stable, fine-scale geographic resolution comparable to census tracts in the United States or dissemination areas in Canada.

### Study sample

The unit of analysis was the 2011 small-area geography comprised of Lower Layer Support Output Areas (LSOAs) in England and Wales and Data Zones (DZs) in Scotland. From a maximum of 47,422 small-area units (32,844 LSOAs in England, 1,909 LSOAs in Wales, and 12,669 DZs in Scotland), a harmonized subset of 41,729 small areas had complete data on food desert risk and deprivation.

### Primary outcome: food desert index (EFDI)

The EFDI reflects barriers to affordable and nutritious food and includes two domains: Access to Food Retail (Physical Accessibility: network‑based distance/travel time to major supermarkets and grocery retailers, and density/proximity of outlets) and Food Affordability and Socioeconomic Risk (Financial Accessibility: household income, car ownership/transport availability, and digital access relevant to online grocery procurement) [26]. The EFDI scores are standardized and combined into an overall index score, with higher values indicating greater risk of food desert conditions [26]. The final EFDI scores are ranked and scaled within each country to maintain internal comparability [26].

In this study, the EFDI was tested against small-area deprivation indices, mainly the IMD for England, the WIMD for Wales and the SIMD for Scotland, and geographic boundaries at the LSOA/Data Zone level using standardized 2011 Census codes [25]. The EFDI values were normalized across all three countries to ensure cross-national comparability [26]. Areas in the highest quartile of EFDI scores were classified as "high-risk food deserts," while those in the lowest quartile were classified as "low-risk" [26]. These data are now available at: https://data.hasp.ac.uk/browser/dataset/5347/0. Thus, while the goal was to examine the relationship between EFDI and IMD in the UK, EFDI was only measured for Great Britain (England, Wales and Scotland), thus excluding Northern Ireland and limiting the ability to extrapolate our findings to the entirety of the UK.

### Primary exposure: index of multiple deprivation

The IMD is a widely used area-level measure of relative socioeconomic deprivation across the UK [27]. The study used the most recent national IMDs: English IMD (2019), Welsh IMD (2019), and Scottish IMD (2020) [27]. These indices integrate weighted domains including income, employment, education, health, crime, housing, and environment. Each was linked using 2011 Census identifiers (UK LSOA/DZ/SDZ). To align directionality with the EFDI (higher score → more food desert risk) and IMD scores/ranks, IMD overall and component-specific metrics were reverse-coded and standardized where appropriate. Harmonized IMD quartiles were created to classify areas by deprivation severity. The data is now available at: https://data.geods.ac.uk/dataset/index-of-multiple-deprivation-imd.

### Demographic and built environment variables

Another data source was used that combined UK LSOA/DZ/SDZ Classification (2021/2 LSOAC) and included geographical identifiers, classification codes, demographic and socioeconomic indicators, and housing tenure. It also includes demographic and socioeconomic indicators such as population density, household composition, ethnic diversity, age structure, employment status, educational attainment, housing tenure, health indicators, car ownership, urban/rural classification, and built environment and infrastructure. Access to the full dataset requires registration and approval at: https://data.geods.ac.uk/dataset/lsoac. While this dataset is safeguarded, descriptives for the classifications can be found at the same URL. These codes were as follows: 1. Retired professionals (referent), 2. Suburbanites and Peri-Urbanites; 3. Multicultural and educated urbanites, 4. low-skilled migrant and student communities, 5. Ethnically diverse sub-urban professionals, 6. Baseline UK, 7. Semi- and Un-Skilled Workforce, 8. Legacy Communities.

### Statistical methods

Our statistical methods entailed descriptives and spatial distributions with choropleths and k‑means clustering; with primary analyses focused on quantifying associations between IMD and EFDI using Pearson correlation, local polynomial smoothing, multinomial regression (RRR) and OLS regression models. Adaptive least absolute shrinkage selection operator (LASSO) was used to test components of IMD against EFDI (with main effects, and with main effect + two‑way interactions), and sensitivity analyses with mixed‑effects and spatial models. Significance threshold α = 0.05 (two‑sided).

First, descriptive and spatial analyses were conducted at the LSOA/DZ level harmonized EFDI and IMD based on 2011 Census geographic boundaries [25–27]. A categorical typology was created to characterize concordance:

- High-High: Top quartiles (Q3 or Q4) for both EFDI and IMD
- Low-Low: Bottom quartiles (Q1 or Q2) for both indices
- Mixed High: High deprivation (Q3 or Q4) with low food desert risk (Q1 or Q2)
- Mixed Low: Low deprivation (Q1 or Q2) with high food desert risk (Q3 or Q4).
- No data: There is no data available on food desert risk or IMD levels for the LSOA/DZ.

Summary statistics were calculated overall and by country. Geographic data were merged with 2011 Census shapefiles, and choropleth maps (a thematic map where areas are shaded or patterned in proportion to a measurement of a statistical variable) were generated using geographic information system (GIS) tools in Stata (version 18.0; StataCorp) to illustrate the spatial distribution and concordance between EFDI and IMD quartiles patterns across Great Britain. Clusters were color-coded as follows: High-High (red), Low-Low (blue), Mixed (gray), and missing (white). Area (km^2^) was summarized by nation across food desert risk quartiles, deprivation quartiles, and joint EFDI–IMD clusters. Mean area was compared using one-way ANOVA, with Bonferroni correction for multiple testing. K-means clustering (K = 4) a method of vector quantization, originally used for signal processing, was applied to each of the z-scored EDFI across all LSOA/DZs in Great Britain (Mean for raw score in Great Britain was 21.66; 1 SD ~ 10.96) and IMD values. Associations were further evaluated between EFDI and IMD using the Pearson correlation coefficient. Locally weighted scatterplot smoothing was used to assess the shape of the EFDI-IMD relationship overall and within each country. To evaluate relationships amongst categorical variables, a multinomial logistic regression model was estimated, with IMD quartile membership as the dependent variable and EFDI quartile as the independent variable. Relative risk ratios (RRRs) and 95% confidence intervals (CIs) were reported to quantify the association between food environment disadvantage and deprivation category membership.

Second, to examine each IMD component in relation to food desert risk, a set of multivariable-adjusted ordinary least square models were used with food desert risk as the main continuous outcomes, and each available and harmonized key IMD component (education, income, employment, crime, housing, access to services, and health) as an independent predictor.

We further implemented a series of machine-learning models using the Least Absolute Shrinkage and Selection Operator (LASSO), a prediction tool that identifies stable correlates under penalization. Models were estimated separately for England, Scotland, and Wales using 6–8 z-scored IMD components as predictors and the z-scored food desert risk score (1 SD = 10.95 across Great Britain) as the outcome. LASSO applies an L1 penalty that shrinks weaker predictors toward zero, facilitating variable selection in the presence of correlated covariates. Within each nation, data were randomly split into training and validation subsets to tune the regularization parameter (λ) via cross-validation and minimum BIC criteria (Appendix I), and the selected model was then refit to the full national sample. Hierarchical LASSO models incorporating two-way interaction terms under the strong heredity principle were additionally estimated to explore synergistic or antagonistic effects. R-squared estimates were evaluated in each model, to assess overall fit and the relative contribution of fixed effects to explaining outcome variability.

Robustness was assessed using linear mixed-effects models with random intercepts for local authorities and, for England and Wales, models further adjusted for area classification to evaluate sensitivity of main and interaction effects. In mixed-effects linear regression models, variance components and the intracluster correlation were provided to assess In mixed-effects linear regression models, variance components and the intracluster correlation were provided to assess the degree of outcome clustering and the proportion of total variance attributable to between-cluster differences.

It is worth noting that IMD component measures differ across nations: England and Wales report deprivation as scores (higher values indicate greater deprivation), whereas Scotland uses ranks, where lower values indicate greater deprivation. Although components were standardized and reverse-coded to align directionality, scores and ranks capture deprivation differently. Accordingly, coefficients should be interpreted primarily in terms of direction and within-nation patterns, rather than as directly comparable effect sizes across countries.

Third, to examine spatial patterns and associations between neighborhood deprivation and food access, we utilized *GeoDa 1.22.0.21 *software (https://geodacenter.github.io/) to conduct univariate and bivariate spatial analyses. A bivariate spatial autocorrelation estimate (Moran’s I) was generated with the two variables being z-scored Food Desert Index (zFD) and IMD (zIMD) at the LSOA 2011 level, using a spatial weight matrix constructed from 4 nearest neighbors (kNN). We then estimated a bivariate regression model with zFD as the dependent variable and z-scored Index of Multiple Deprivation (zIMD) as the predictor, and vice versa as a sensitivity analysis, also using a 4-kNN spatial weights matrix. Shapefiles for the LSOA 2011 boundaries were sourced from the UK Office for National Statistics. Most analyses were conducted in Stata, with parts done in R version 4.5.2 and GeoDA version 1.22.0.21, and statistical significance was assessed using a 2-sided α = 0.05.

## Results

The analytic sample included 41,729 geographic units across the UK, with 34,753 in England and Wales and 6,976 in Scotland (Table 1). Food desert risk scores were slightly higher in Scotland (mean, 23.7) versus England and Wales (mean, 21.3), with standardized scores reflecting a similar trend. Risk increased across deprivation quartiles, with a more pronounced gradient in Scotland. IMD was similarly distributed across regions. Cluster analysis showed that most areas were classified as “low-low” (31.8%) or “mixed-high” (32.3%), with Scotland having more “low-low” and fewer “mixed-low” areas than England and Wales.

**Table 1:** Study sample characteristics at the LSOA/DZ level, UK 2015-2019^a^

	**Overall**	**England & Wales**	**Scotland**
	***N* = 41,729**	**N = 34,753**	***N* = 6,976**
Food desert risk			
Raw: Mean ± SD	21.7 ± 11.0	21.3 ± 10.3	23.7 ± 13.7
z-score: Mean ± SD	0.00 ± 1.00	−0.036 ± 0.94	0.18 ± 1.25
Means ± SD within quartiles			
Q1	−1.09 ± 0.28	−1.07 ± 0.26	−1.19 ± 0.33
Q2	−0.44 ± 0.15	−0.44 ± 0.15	−0.45 ± 0.15
Q3	−0.15 ± 0.21	+ 0.15 ± 0.21	+ 0.17 ± 0.21
Q4	1.38 ± 0.73	+ 1.32 ± 0.65	+ 1.58 ± 0.94
Index of multiple deprivation, IMD, harmonized			
Raw: Mean ± SD	50.00 ± 28.87	50.00 ± 28.87	50.00 ± 28.87
z-score, reverse coded: Mean ± SD, overall	+ 0.00 ± 1.00	+ 0.00 ± 1.00	−0.00 ± 1.00
Means ± SD within quartile			
Q1	−1.29 ± 0.26	−1.29 ± 0.26	−1.29 ± 0.26
Q2	−0.42 ± 0.25	−0.42 ± 0.25	−0.42 ± 0.25
Q3	+ 0.45 ± 0.25	+ 0.45 ± 0.25	+ 0.45 ± 0.25
Q4	+ 1.30 ± 0.25	+ 1.31 ± 0.25	+ 1.31 ± 0.25
Clustering of IMD and Food desert risk	N(%)	N(%)	N(%)
Low-Low	13,276 (31.8)	10,781 (31.0)	2,495 (35.8)
Mixed-High	13,487 (32.3)	11,278 (32.5)	2,209 (31.7)
Mixed-Low	7,378 (17.7)	6,420 (18.5)	958 (13.7)
High-High	7,588 (18.2)	6,274 (18.1)	1,314 (18.8)

*DZ* Data Zone, *LSOA* Lower Statistical Output Area, *Q1* First Quartile, *Q2* Second Quartile, *Q3* Third Quartile, *Q4* Fourth Quartile, *SD* Standard Deviation, *UK* United Kingdom

^a^Values are presented as means ± standard deviations (SD) for continuous variables and as absolute frequencies (N) with column percentages (%) for categorical variables. Food desert risk is shown both on its original scale and as a standardized z-score (mean = 0, SD = 1) calculated across all LSOAs/DZs. Index of Multiple Deprivation (IMD) scores were harmonized across England, Wales, and Scotland and reverse coded such that higher z-scores indicate greater socioeconomic deprivation. Quartiles (Q1–Q4) were defined based on the national distributions of the corresponding z-scored measures across all areas

**Fig. 1:**
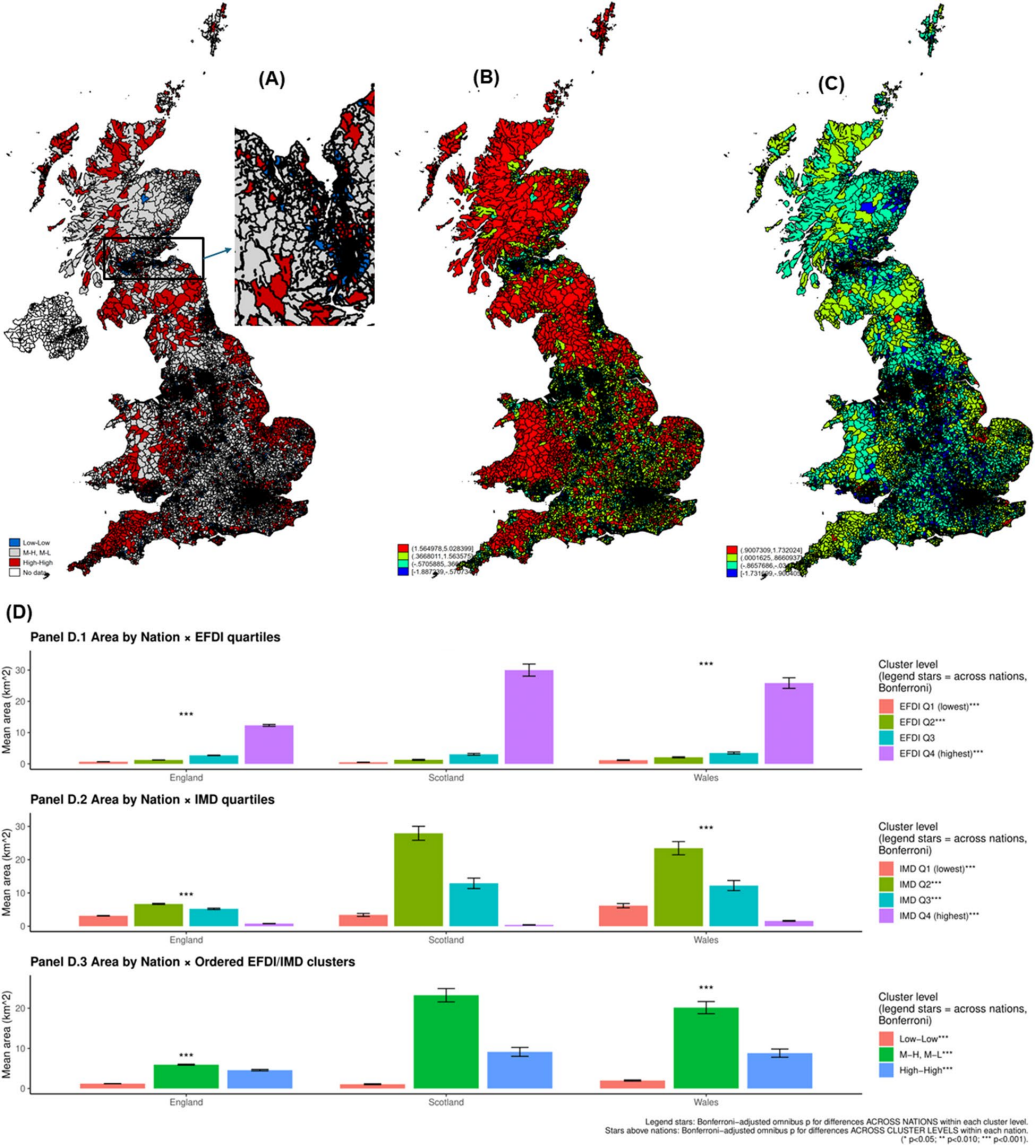
Spatial distribution and clustering of food desert risk and area-level deprivation across Great Britain (LSOA/Data Zone level), 2015–2019. **A** Joint classification of food desert risk and Index of Multiple Deprivation (IMD) using quartiles of both standardized measures. Areas are grouped into concordant and discordant categories: High–High (high food desert risk and high deprivation; red), Low–Low (low food desert risk and low deprivation; blue), Mixed (high–low or low–high combinations; gray), and No data (white). This panel highlights spatial concordance and mismatch between food access disadvantage and deprivation. **B** K-means clustering (k = 4) of the standardized food desert risk (EFDI) score, applied independently of deprivation, illustrating data-driven spatial groupings of food access risk across Great Britain. **C** K-means clustering (k = 4) of standardized IMD scores (reverse-coded where applicable), illustrating the spatial distribution of deprivation independent of food desert risk. **D** Average area (km.^2^) by nation across food desert risk quartiles, deprivation (IMD) quartiles, and ordered joint EFDI–IMD clusters, with Bonferroni-adjusted tests for differences across nations and within nations across cluster levels. *Abbreviations*: DZ = Data Zone; IMD = Index of Multiple Deprivation; LSOA = Lower Layer Super Output Area; UK = United Kingdom. *Sources*: [25–27]

Geospatial distribution of IMD and food desert risk z-scores across LSOA/DZ are presented in Fig. 1. K-means clustering was used as an unsupervised, data-driven method to visualize FD risk and IMD distributions separately, while quartiles were used to define comparative cluster groupings across the two measures, as shown in Fig. 1A-D. Clustering values between those two variables using quartiles yielded four combinations of levels for food desert and deprivation indices. Frequencies were relatively evenly distributed: 31.8% of LSOAs/DZs were in the “High-High” cluster, 32.3% in the “Low-Low,” and the remainder in mixed categories —“Mixed High” (17.7%) and “Mixed Low” (18.2%). Figure 1 (Panel A) also presents those clusters, focusing on High-High (red) and Low-Low (blue) categories. Based on this Figure, larger LSOAs/DZs with a similar number of inhabitants (1000–3000 per area) tended to experience a High-High concordance between food desert and area deprivation status. In contrast, most Low-Low areas were smaller areas with similar numbers of inhabitants, suggesting suburban or urban areas, with concomitantly greater wealth, more resources, and access to healthy food options. Across all panels D.1 through D.3 of Fig. 1, mean area differed markedly by nation and deprivation context. In Panel D.1, higher EFDI quartiles were consistently associated with substantially larger geographic areas, particularly in Scotland and Wales, with significant differences across nations and within nations across quartiles. Panel D.2 shows a complementary pattern for IMD, where mid-to-higher deprivation quartiles corresponded to larger areas, again most pronounced in Scotland and Wales. Panel D.3 demonstrates that ordered joint EFDI–IMD clusters capture these gradients: mixed and high–high clusters encompassed significantly larger areas than low–low clusters, with strong cross-national and within-nation heterogeneity after Bonferroni correction. Details of these findings are provided in supplementary datasheet 1.

**Fig. 2:**
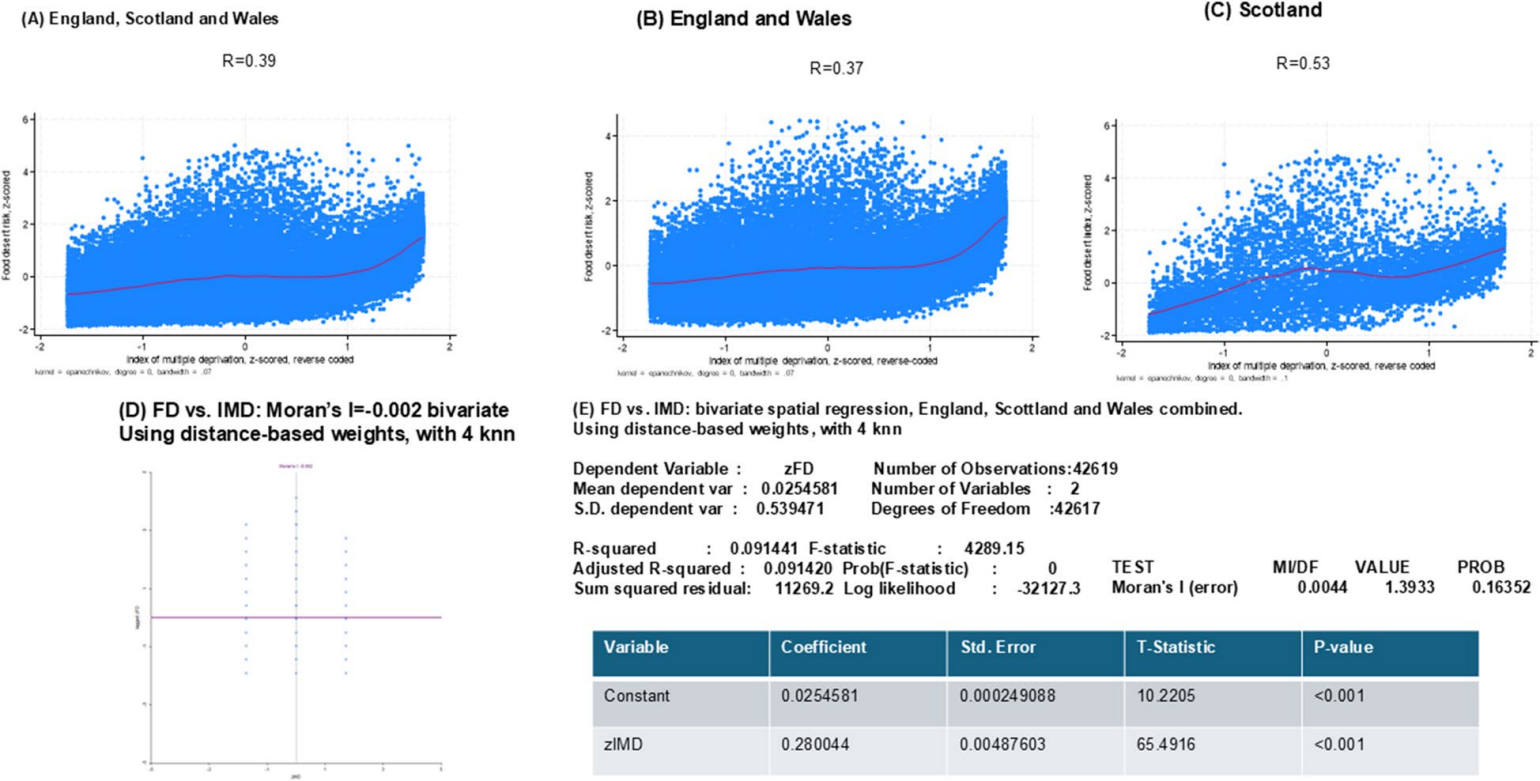
Association and spatial dependence between food desert risk and area-level deprivation at the LSOA/Data Zone level, Great Britain, 2015–2019. **A–D** Local polynomial smoothing plots showing the bivariate association between food desert risk and deprivation for Great Britain overall and stratified by England, Wales, and Scotland, respectively. Curves depict the average trend in food desert risk across the deprivation distribution, highlighting nonlinearity and cross-national differences in gradients. **E** Results from bivariate spatial regression models using a 4-nearest-neighbor spatial weights matrix. When IMD is specified as the predictor of food desert risk, residual Moran’s I is small and non-significant, indicating that the spatial specification adequately accounts for spatial autocorrelation. In contrast, residual spatial autocorrelation persists when food desert risk is specified as the predictor of IMD, suggesting broader spatial structuring of deprivation not fully captured by food access alone. *Abbreviations*: DZ = Data Zone; IMD = Index of Multiple Deprivation; LSOA = Lower Layer Super Output Area; UK = United Kingdom. *Sources*: [25–27]

Cross-tabulation of deprivation and food desert quartiles demonstrated a strong association (Pearson χ^2^(9) = 7,900; *p* < 0.001), with nearly half (48.2%) of most deprived areas (Q4) also in the highest food desert quartile. Using linear regression, the deprivation continuous z-score (reverse coded) was positively associated with food desert z-score (β = 0.396, 95% CI: 0.387, 0.405; *p* < 0.001), explaining 15.7% of the variance in food desert exposure (adjusted R^2^ = 0.1566). Pearson’s correlation (r ~ 0.40, *P* < 0.001) and local polynomial smoothing of z-scores for the two variables are presented in Fig. 2. In panel E of Fig. 2, bivariate spatial regression showed a significant positive association between zIMD and zFD (β = 0.280, *p* < 0.001), indicating that greater deprivation is associated with higher food desert scores. The model explained approximately 9.1% of the variance (Adjusted R^2^ = 0.091). Residual Moran’s I was small and not statistically significant when IMD was modeled as a predictor of EFDI z-score (I = 0.0044, *p* = 0.16), indicating that the spatial specification adequately accounted for spatial dependence. In contrast, residual spatial autocorrelation remained when EFDI z-score was used to predict IMD (I = 0.0116, *p* < 0.001), suggesting that deprivation exhibits broader spatial structure not captured by food access alone. These results support the chosen spatial model and the primary analytic direction.

Based on this Fig. 2 and Table 1, concordance between IMD and food desert risk was higher in Scotland compared with England and Wales combined. Multinomial logistic regression showed a strong, graded relationship between food desert quartile and deprivation category (e.g., RRR for highest food desert risk vs. lowest per quartile increase in IMD (z-scored, reverse coded): RRR = 3.07, 95% CI: 2.98–3.16, *p* < 0.001), as shown in Table 2.

**Table 2:** Multinomial logit regression model for quartiles of index of multiple deprivation (z-scored, reverse-coded) vs. quartiles of food desert risk (z-scored) at the LSOA/DZ levels: UK 2015-2019^a^

	**Y = Food desert risk, z-scored**			
	**Q1**	**Q2**	**Q3**	**Q4**
	Mean ± SD	Mean ± SD	Mean ± SD	Mean ± SD
	−1.090 ± 0.275	−0.442 ± 0.152	+ 0.149 ± 0.207	+ 1.383 ± 0.733
	RRR with 95% CI			
X = Index of multiple deprivation, quartiles				
Model 1				
Index of multiple deprivation				
Quartile, ordinal	1.00	1.63 (1.58; 1.67)***	2.26(2.19; 2.32)***	3.07 (2.98; 3.16)***
Model 2				
Index of multiple deprivation				
Quartile, categories				
Q1: −1.290 ± 0.255	1.00	1.00	1.00	1.00
Q2: −0.415 ± 0.250	1.00	1.19 (1.11; 1.27)***	1.49 (1.38; 1.60)***	3.28 (3.01; 3.58)***
Q3: + 0.450 ± 0.250	1.00	2.21 (2.06;2.37)***	3.07 (2.84; 3.31)***	4.33 (3.95; 4.74)***
Q4: + 1.308 ± 0.245	1.00	8.15 (7.17; 9.26)***	23.63 (20.82; 26.82)***	70.67(61.91; 80.67)***

*CI* Confidence Interval, *DZ* Data Zone, *LSOA* Lower Statistical Output Area, *Q1* First Quartile, *Q2* Second Quartile, *Q3* Third Quartile, *Q4* Fourth Quartile, *RRR* Relative Risk Ratio, *SD* Standard Deviation, *UK* United Kingdom

^a^Multinomial logit regression models were estimated at the LSOA/DZ level with quartiles of food desert risk (z-scored; Q1 as the reference category) as the outcome. Index of Multiple Deprivation (IMD) was harmonized across England, Wales, and Scotland, reverse coded such that higher values indicate greater socioeconomic deprivation, and standardized (z-score). Model 1 treated IMD quartiles as an ordinal predictor to estimate a linear trend across deprivation levels, whereas Model 2 included IMD quartiles as categorical predictors (Q1 as the reference). Relative risk ratios (RRRs) and 95% confidence intervals (CIs) are reported. Quartiles were defined based on national distributions of the corresponding z-scored measures across all LSOAs/DZs

^*^ *P* < 0.05 indicates rejection of the null hypothesis that the relative risk ratio equals 1.00 at the 5% significance level;

^**^ *P* < 0.010 indicates rejection of the null hypothesis at the 1% significance level;

^***^ *P* < 0.001 indicates rejection of the null hypothesis at the 0.1% significance level

Sources: [25–27]

LASSO regression model identified significant predictors of food deserts in England, Scotland, and Wales. As noted earlier, higher overall IMD values indicate greater deprivation, but component-level measures are not directly comparable across nations. England and Wales report deprivation as scores (higher values indicating greater deprivation), whereas Scotland uses ranks, requiring inverse interpretation (lower rank = greater deprivation). Based on the adaptive LASSO, which excluded less predictive factors, in all three nations, the employment sub-score was the most predictive of the food desert z-score, closely followed by the health score in Wales, the housing score in Scotland and the crime score in England (Table 3, See Appendix II). The final selected model based on adaptive LASSO is shown in Table 3, for each of the 3 nations, using z-scored sub-domains and z-scored food desert outcome. Specifically, in these regression models across England, Scotland, and Wales, employment deprivation consistently emerged as the strongest predictor of food desert risk, with education deprivation being also positively associated, though to a lesser extent. Unexpectedly, health deprivation showed an inverse relationship, with poorer health areas less likely to be food deserts. However, mixed-effect models indicated that this effect was no longer detected in Wales, after accounting for clustering at the local authority level. In England, additional predictors such as housing/access to services and the living environment were positively associated, while crime was inversely related. Scotland's housing deprivation rank also predicted higher food desert risk. These findings highlight employment as a key structural determinant of food access, with notable variation across deprivation domains and national contexts.

**Table 3:** Ordinary Least Square regression model for selected components of multiple deprivation (z-scored, reverse-coded) vs. food desert risk (z-scored) at the LSOA/DZ levels for each nation: UK 2015–2019

	***Y = Food desert risk, z-scored***		
	***England***	***Scotland***	***Wales***
	*β ± SE*	*β ± SE*	*β ± SE*
	***Score***	***Rank***	***Score***
	***↑ score ↑ deprivation***	***↑ rank ↓ deprivation***	***↑ score ↑ deprivation***
	***N*** *** = 32,844***	***N*** *** = 6,976***	***N*** *** = 1,909***
X = Component of multiple deprivation, z-scored^a^			
OLS			
Income score/rank			
Employment score/rank	+ 0.621 ± 0.010***	−0.459 ± 0.027***	+ 0.662 ± 0.065***
Education score/rank	+ 0.165 ± 0.007***	−0.277 ± 0.028***	+ 0.127 ± 0.052*
Health score/rank	−0.173 ± 0.008***	…	−0.501 ± 0.072***
Housing score/rank	…^b^	+ 0.380 ± 0.019***	…
Crime score/rank	−0.391 ± 0.005***	…	…
Access to services score/rank	…^b^	…	…
Housing and access to service score/rank	+ 0.214 ± 0.004***	…	…
Living Environment	+ 0.079 ± 0.004***	…	…
Intercept	−0.058 ± 0.004***	+ 0.181 ± 0.014***	+ 0.368 ± 0.028***
R-squared	0.412	0.171	0.083
Mixed-effects models with LA random effect for intercept^c^			
Income score/rank	…	…	…
Employment score/rank	+ 0.633 ± 0.005***	−0.384 ± 0.023***	+ 0.395 ± 0.050***
Education score/rank	+ 0.054 ± 0.007***	−0.198 ± 0.024***	+ 0.079 ± 0.036
Health score/rank	−0.205 ± 0.009***	…	−0.022 ± 0.055
Housing score/rank	…^b^	+ 0.116 ± 0.011***	…
Crime score/rank	−0.192 ± 0.005***	…	…
Access to services score/rank	…^b^	…	…
Housing and access to service score/rank	+ 0.312 ± 0.004***	…	…
Living Environment	+ 0.102 ± 0.004***	…	…
Intercept	+ 0.041 ± 0.029***	+ 0.486 ± 0.167***	+ 0.536 ± 0.188**
Number of clusters	317	32	22
Var (_cons)	0.259 (0.021)	0.883 (0.225)	0.764 (0.233)
Var (Residual)	0.298 (0.002)	0.837 (0.014)	0.786 (0.026)
ICC	0.46	0.51	0.49

*CI* Confidence Interval, *DZ* Data Zone, *ICC* Intra-cluster correlation, *IMD* Index of Multiple Deprivation, *LA* Local Authority,* LASSO* Least Absolute Shrinkage Selection Operator, *LSOA* Lower Statistical Output Area, *SE* Standard Error, *UK* United Kingdom

^a^Components of the Index of Multiple Deprivation (IMD) were modeled using domain-specific scores for England and Wales and ranks for Scotland, reflecting differences in national IMD construction. In all cases, predictors were standardized as z-scores prior to analysis, and higher values correspond to greater socioeconomic deprivation. For England, the crime domain score was natural-log transformed after adding a constant of 10 to avoid negative values and then z-score standardized. Ordinary least squares models were specified using a data-driven adaptive LASSO variable selection procedure to identify the subset of IMD components independently associated with food desert risk

^b^For England, the housing and access-to-services domains were combined into a single composite measure, consistent with the national IMD framework; therefore, these components were not entered as separate predictors in England-specific models

^c^As a sensitivity analysis, linear mixed-effects regression models were estimated with a random intercept for local authority (LA) to account for clustering of LSOAs/DZs within LAs. Variance components were used to quantify between- and within-LA variability, and the intracluster correlation coefficient (ICC) was calculated to assess the proportion of total variance in food desert risk attributable to differences between local authorities

^*^ P < 0.05 indicates rejection of the null hypothesis that the regression coefficient equals zero at the 5% significance level;

^**^ P < 0.010 indicates rejection of the null hypothesis at the 1% significance level;

^***^ P < 0.001 indicates rejection of the null hypothesis at the 0.1% significance level

Sources: [25–27]

Based on hierarchical adaptive LASSO models across England, Scotland, and Wales, food desert risk was associated with overlapping but context-specific patterns of area-level deprivation (Table 4; Table S1). Across nations, employment deprivation consistently showed the strongest association with food desert risk, with additional contributions from income, housing/access to services, and the living environment. Crime and health deprivation were inversely or inconsistently associated, and patterns differed by national IMD construction, particularly in Scotland where deprivation is rank-based rather than score-based. Although several two-way interaction terms reached statistical significance, these findings were heterogeneous across countries and model specifications. To maintain interpretability, emphasis is placed on interactions involving employment deprivation, which were the most recurrent and policy relevant. Interactions involving employment deprivation were most consistently selected. Employment × Housing/Access to Services interactions suggested compounding structural barriers in England, whereas the antagonistic Employment × Income interaction likely reflects shared variance between economic deprivation domains rather than synergistic effects in all Great Britain. These interactions aligned with the strong main effects of employment deprivation observed across all three nations. Most other interaction terms varied in direction across countries, lost significance in mixed-effects models accounting for local authority clustering, or were sensitive to national differences in IMD operationalization (Table S1).

**Table 4:** Ordinary Least Square regression model for selected components and their selected 2-way interaction terms vs. food desert risk (z-scored) at the LSOA/DZ levels for each nation: UK 2015-2019

	**Y=Food desert risk, z-scored**		
	**England**	**Scotland**	**Wales**
	β±SE	β±SE	β±SE
	**Score**	**Rank**	**Score**
	**↑ score ↑ deprivation **	**↑ rank ↓ deprivation**	**↑ score ↑ deprivation**
	***N*=32,844**	***N*=6,976**	***N*=1,909**
X=Component of multiple deprivation and their 2-way interactions, z-scored^a^			
Main effects			
Income score/rank	-0.239±0.018***	-0.487±0.050***	+0.416±0.073***
Employment score/rank	+0.729±0.018***	-0.308±0.045***	+0.641±0.059***
Education score/rank	+0.174±0.009***	-0.073±0.026**	…
Health score/rank	-0.105±0.008***	…	-0.510±0.061***
Housing score/rank	…^b^	+0.138±0.019***	+0.118±0.029***
Crime score/rank	-0.266±0.005***	+0.231±0.016***	-0.328±0.033***
Access to services score/rank	…^b^	-0.497±0.013***	+0.576±0.027***
Housing and access to service score/rank	+0.165±0.014***	…	…
Living Environment	+0.047±0.005***	…	-0.010±0.001***
Two-way interaction terms			
Health×Income	+0.343±0.022***	…	…
Housing/service×Income	-0.184±0.013***	…	…
Education×Health	-0.158±0.015***	…	+0.064±0.021**
Employment×Income	-0.117±0.012***	+0.161±0.033***	-0.308±0.044***
Crime×Income	+0.164±0.018***	-0.005±0.058	…
Employment×Housing/service	+0.166±0.014***	…	…
Education×Employment	+0.109±0.018***	*+0.103±0.058*	…
Crime×Living environment	-0.136±0.005***	…	+0.002±0.001*
Health×Housing/service	-0.156±0.008***	…	
Crime×Employment	-0.107±0.019***	*-0.095±0.051*	…
Housing/service×Living environment	+0.116±0.004***	…	…
Employment×Health	-0.099±0.019***	…	+0.188±0.046***
Income×Living environment	-0.132±0.015***	…	…
Employment×Living environment	+0.104±0.014***	…	…
Education×Living environment	+0.081±0.007***	…	…
Eduction×Income	-0.044±0.016***	-0.186±0.058***	
Crime× Housing/service	-0.042±0.005***	…	…
Crime×Health	+0.046±0.010***	…	+0.116±0.021***
Health×Living environment	-0.026±0.008***	…	…
Education×Housing/services	+0.025±0.008**	…	…
Crime×Education	-0.024±0.010*	+0.150±0.031***	…
Service×Income	…	-0.261±0.017***	…
Service×Employment	…	-0.111±0.045*	…
Service×Housing	…	+0.112±0.018***	…
Crime×Housing	…	-0.119±0.021***	-0.034±0.021***
Service×Income	…	+0.002±0.051	…
Service×Education	…	*-0.049±0.028*	…
Housing×Income	…	+0.019±0.032	+0.023±0.022
Service×Health	…	…	-0.192±0.036***
Service×Crime	…	-0.261±0.016***	-0.126±0.031***
Housing×Living environment	…	…	-0.003±0.001***
Education×Housing	…	-0.162±+0.030***	…
Intercept	-0.083±0.005***	+0.125±0.018***	+0.582±0.031***
R-squared	0.511	0.433	0.663

*CI* Confidence Interval, *DZ *Data Zone, *IMD *Index of Multiple Deprivation, *LASSO *Least Absolute Shrinkage Selection Operator, *LSOA *Lower Statistical Output Area, *SE *Standard Error, *UK *United Kingdom

^a^Components of the Index of Multiple Deprivation (IMD) were modeled using domain-specific scores for England and Wales and ranks for Scotland, reflecting differences in national IMD construction. In all cases, predictors were standardized as z-scores prior to analysis, such that higher values indicate greater socioeconomic deprivation. For England, the crime domain score was natural-log transformed after adding a constant of 10 to avoid negative values and subsequently z-score standardized. Ordinary least squares regression models were estimated using a hierarchical adaptive LASSO variable-selection procedure that simultaneously considered all available IMD main effects and all possible two-way interaction terms, retaining only those terms independently associated with food desert risk

^b^For England, the housing and access-to-services domains were combined into a single composite measure in accordance with the national IMD framework; therefore, these domains were not modeled as separate predictors in England-specific analyses

* P < 0.05 indicates rejection of the null hypothesis that the regression coefficient equals zero at the 5% significance level

** P < 0.010 indicates rejection of the null hypothesis at the 1% significance level

*** P < 0.001 indicates rejection of the null hypothesis at the 0.1% significance level

Sources: [25–27]

Mixed-effects models generally produced more conservative estimates than ordinary least squares models, underscoring the importance of accounting for geographic nesting and spatial heterogeneity. Further adjustment for LSOA-level socio-demographic classifications in England and Wales did not materially alter the primary associations (Table S2). Sample sizes differ between Table S1 and Table S2 because the latter adjusts for an LSOA-level socio-demographic classification available only for England and Wales, leading to exclusion of areas with missing classification data (e.g., England: 32,844 vs. 31,810; Wales: 1,909 vs. 1,837). These differences reflect data availability rather than selective exclusion and may affect comparability across models. Finally, across analyses, OLS and LASSO models explained substantially more variance in food desert risk (R^2^ ≈ 0.41 in England, 0.17 in Scotland, 0.08 in Wales) than the spatial regression model (adjusted R^2^ ≈ 0.09). Mixed-effects models revealed moderate-to-high clustering at the local-authority level (ICC ≈ 0.33–0.51), indicating meaningful geographic nesting. Together, these findings suggest that deprivation components drive most explainable variation, while spatial structure and clustering shape residual heterogeneity rather than primary associations.

## Discussion

​ Across 41,729 small areas, deprivation and EFDI were moderately correlated (r = 0.40), with nearly half of the most deprived areas (Q4) also in the highest EFDI quartile. Employment deprivation consistently predicted higher food desert risk across nations, while education, housing/access to services, living environment, and crime showed nation-specific patterns. The two indices share structural determinants of health but capture overlapping yet distinct dimensions of disadvantage. Employment deprivation emerged as being strongly linked to limited food access, underscoring its central role in shaping community food environments. Large rural areas with low overall deprivation but high food desert risk may be overlooked by policies relying solely on deprivation indices. Results emphasize the multifactorial and interactive nature of food access inequities. Spatial analyses further supported IMD as likely associated with food desert patterns, given minimal residual autocorrelation when IMD predicted EFDI but not the reverse.

Prior research has consistently shown that food deserts—areas with limited access to affordable, nutritious food—tend to cluster in socioeconomically disadvantaged neighborhoods [5–7, 11]. Lower-income areas are less likely to host large supermarkets, in part due to crime rates that disincentivize investments, and are more likely to rely on convenience stores, which often offer fewer and lower-quality healthy options [5, 28]. Consequently, lower-income individuals may end up spending more than higher-income individuals on lower-quality groceries. However, our findings, together with prior work [6, 12–20], suggest that food access does not follow a simple deprivation gradient. The inverse associations observed for domains such as health and crime likely reflect urban concentration and legacy infrastructure, where highly deprived areas retain dense retail and service networks, while less deprived but more rural or peri-urban areas face greater physical access barriers. In a recent study, a non-linear socioeconomic inequality in the spatial distribution of food outlets was observed across metropolitan Melbourne and regional Victoria, Australia [11]. Therefore, both the most disadvantaged and most advantaged areas had higher densities of food outlets, while mid-socioeconomic areas were under-served [11]. Patterns persisted across healthy, less healthy, and unhealthy outlet types, suggesting complex, non-monotonic associations between area deprivation (similar to IMD) and food environment characteristics (FD) [11].

This non-linearity has been detected in other studies as well based on a recent review [6, 12–20]. Based on this review, across North America for instance, these studies show that relationships between neighborhood SES, race/ethnicity, and food access are context-dependent and highly sensitive to measurement [12]. Several studies report limited or absent “classic” food deserts in dense urban areas (e.g., Montréal, NYC), while others find reduced supermarket proximity and healthy food access in poorer or minority neighborhoods, especially in suburban or rural settings [6, 12–20]. Results vary by metric (distance vs density), buffer choice, and outlet definition, producing sometimes non-linear or mixed gradients rather than a simple SES–food desert relationship [12].

Disadvantaged small geographical areas often experience co-occurring food access challenges, including elevated food desert risk and food insecurity; however, the E-Food Desert Index (EFDI) captures area-level structural barriers to food access (e.g., physical and financial accessibility) and does not measure household-level food insecurity, diet quality, or health outcomes, which were not directly assessed in the present analysis. Prior studies suggest that economic factors such as unemployment are associated with higher food insecurity risk, with repeated episodes compounding vulnerability [29]. Food insecurity and neighborhood safety have been shown to co-occur with food access disparities in prior work, but these relationships remain hypotheses in the context of the current area-level study and warrant investigation using multilevel designs. Evidence from external studies indicates that unemployment support and other forms of financial assistance may be associated with lower food insecurity [30]. More broadly, access to healthy food reflects a complex interplay of individual and environmental factors [5], including household constraints (e.g., single parenthood, inflexible work schedules, limited transportation) and neighborhood characteristics such as public transit availability and perceived safety.

Crime represents another contextual factor that has been linked to food security and diet quality in prior research, though it was not directly examined in this study. Victimization has been associated with poorer diet and greater food insecurity [31, 32], and some literature suggests potentially bidirectional relationships between nutrition, neurocognitive development, and criminal behavior [32]. Neighborhood-level studies have reported that food-insecure households perceive lower safety and cohesion, findings that align with observed correlations between food insecurity and violent crime in administrative data. These pathways are discussed here as contextual background rather than empirically supported mechanisms within the current analysis. Given these interconnections, identifying effective interventions remains challenging. Nonetheless, prior evidence suggests that nutrition and financial assistance programs are associated with improvements in food security, and participation in the U.S. Department of Agriculture Supplemental Nutrition Assistance Program (SNAP) has been linked in external studies to better diet quality and lower food insecurity, with some evidence of associations with reduced crime [30, 32].

In the UK context, analogous mechanisms may operate through programs such as Healthy Start, Free School Meals, Holiday Activities and Food (HAF) programs, and local welfare assistance schemes, which aim to buffer economic shocks and improve access to nutritious food among vulnerable households [33–35]. Evaluations of UK food support initiatives and food bank use suggest that income instability, benefit adequacy, and local safety conditions are tightly interwoven with food insecurity risk, particularly in deprived neighborhoods [33–35]. However, the extent to which such programs modify neighborhood-level crime or food environment patterns remains insufficiently studied and warrants future multilevel investigation.

Although several studies have examined the spatial distribution of food retail access or area-level deprivation independently, fewer have explicitly linked validated indices of food desert risk to nationally harmonized deprivation metrics at small-area levels. A systematic review by Beaulac et al. highlighted this gap, calling for more robust, geographically nuanced research [8]. More recent efforts, as was done in the current study, attempt to fill this void by combining travel time, income, and vehicle access into a multidimensional index of food access disadvantage, such as the development of the E-Food Desert Index [26].

The relationship between food deserts and chronic disease burden is multifactorial, involving socioeconomic deprivation, food insecurity, unmet healthcare needs, and broader social and environmental influences [5, 7]. Food deserts tend to be concentrated in low-income, rural, and less-educated communities where residents struggle to access affordable and nutritious food [5, 7]. This lack of access contributes to poor diet quality, food insecurity, and a higher prevalence of chronic conditions, including metabolic disorders [36, 37]. Testa et al. (2020) found that food deserts adversely affect cardiovascular health, particularly among socioeconomically marginalized groups [36]. Morris et al. (2019) reported that living in food deserts increases the risk of all-cause and heart failure-related hospitalizations [38]. Suarez et al. (2015), using NHANES data, linked food desert residence to poor diet quality and increased risk of chronic kidney disease [39]. Similarly, Wood et al. (2023) found that food desert exposure worsens metabolic health in pregnant women, with sociodemographic factors compounding risks [40]. Vulnerable populations, including children and older adults, are especially impacted. Francis et al. (2022) found children in food deserts face higher risks of developmental delays and long-term health problems due to poor nutrition and limited access to healthcare [41]. Effective interventions must be multifaceted. Community-level solutions include mobile food vans, farmers markets, and incentives for supermarkets [42, 43]. Educational initiatives—such as cooking classes and community gardens—can empower healthier choices [42, 43]. Policy solutions should prioritize "health in all policies," integrating health considerations into urban planning, housing, and agricultural policies to improve diet and food access [42, 43]. Despite growing interest in place-based health risk factors, national-level analyses integrating food environment metrics with standardized measures of deprivation remain limited. This study contributes to the literature by evaluating the spatial concordance between two conceptually related but methodologically distinct measures across Great Britain. The moderate concordance observed on both continuous and categorical scales highlights the need to consider food environment metrics alongside traditional deprivation indices when assessing local health-related needs. Areas with discordant classifications, such as high food desert risk but low deprivation, may be overlooked in current resource allocation strategies. Our findings also indicate that food desert risk and deprivation are strongly patterned by geographic scale, with higher-risk and mixed EFDI–IMD contexts encompassing substantially larger areas, especially in Scotland and Wales. This spatial expansion suggests that structural barriers to food access in less dense or more rural settings may differ fundamentally from those in compact urban areas. The results underscore the importance of accounting for area size and national context when interpreting food environment inequalities and designing place-based interventions.

This study provides a comprehensive, small-area spatial assessment of food desert risk and socioeconomic deprivation across Great Britain, highlighting the geographic concordance between two major structural determinants of population health. By integrating the English Food Desert Index (EFDI) with the Index of Multiple Deprivation (IMD), the analysis offers a unified framework for characterizing contextual food environments and broader material disadvantage at a national scale.

Several limitations warrant careful consideration. First, as the analysis is conducted at the area level, findings are subject to ecological fallacy; observed spatial associations between EFDI and IMD cannot be assumed to reflect individual-level food access, dietary behaviors, or socioeconomic circumstances. Second, temporal mismatch between underlying datasets may introduce measurement error. Both indices draw on components collected at different time points, and reliance on 2011 Census geographies may not fully capture more recent changes in population distribution, retail landscapes, or transport infrastructure. Third, although EFDI and IMD are standardized metrics, there is substantive conceptual and empirical overlap across several domains, particularly between employment deprivation and EFDI components related to income, transport access, and digital connectivity, which may inflate observed associations. As a result, estimates for employment deprivation should be interpreted as reflecting strong alignment with food desert risk rather than a uniquely independent or causal effect. Full orthogonalization of overlapping domains was not feasible; however, sensitivity analyses using alternative model specifications, including mixed-effects and penalized regression approaches, yielded broadly consistent patterns, supporting the robustness—but not causal interpretation—of these associations. Accordingly, EFDI should be interpreted as a contextual indicator of food accessibility, rather than a causal measure.

Furthermore, additional sources of residual confounding are likely, as both indices omit relevant factors such as food quality, cultural appropriateness, affordability dynamics, informal food sources, and individual mobility. Moreover, formal validation of EFDI against individual-level dietary intake, food insecurity, or health outcomes remains limited. The cross-sectional design further precludes causal inference or assessment of temporal dynamics, and results are inherently scale-dependent, consistent with the modifiable areal unit problem. For instance, findings with employment deprivation being associated with EFDI should be interpreted as a marker of broader structural disadvantage that co-occurs with food access barriers, rather than as an independent causal determinant. Finally, the absence of individual-level behavioral data (e.g., diet, food purchasing, or food security status) restricts insight into the mechanisms linking neighborhood food environments to health.

Despite these limitations, the findings of this study align with a growing literature showing that food desert risk and socioeconomic deprivation are related but not interchangeable dimensions of place-based disadvantage, with important implications for policy targeting. Prior reviews and conceptual work demonstrate that deprivation indices alone do not fully capture food access constraints, particularly when physical accessibility, transport, and retail infrastructure are considered [8, 28]. The strong and consistent association observed between employment deprivation and food desert risk is supported by evidence linking labor market instability to food hardship beyond income alone, reflecting broader structural vulnerabilities related to commuting constraints, service access, and local economic conditions [29, 30]. Associations with housing/access to services and living environment domains further mirror studies emphasizing the role of infrastructure, transport connectivity, and spatial context in shaping food environments, particularly in rural and peri-urban settings [4, 9]. UK-specific research has similarly shown that rural areas may experience substantial food access barriers despite relatively low deprivation rankings, underscoring limitations of deprivation-based targeting alone [26]. Collectively, this evidence supports integrating food environment metrics such as the E-Food Desert Index alongside traditional deprivation measures to refine planning, transport, and public health strategies without implying direct causality.

In conclusion, across Great Britain, food desert risk and socioeconomic deprivation were moderately correlated but remained distinct dimensions of structural disadvantage. Despite a strong graded association, substantial spatial discordance persisted, with many rural areas in Scotland experiencing high food desert risk despite lower overall deprivation. Employment deprivation was the most consistent correlate of food desert risk, with additional contributions from education and housing/access to services and antagonistic employment–income interactions indicating overlap among economic domains. These findings suggest that reliance on deprivation indices alone may miss communities with meaningful food access barriers, and that integrating food environment metrics alongside traditional measures may improve place-based public health and planning efforts.

## Supplementary Information

Supplementary Material 1. Table S1. Mixed-effects linear regression model for selected components and their selected 2-way interaction terms vs. food desert risk (z-scored) at the LSOA/DZ levels for each nation, with random effect added for intercept to account for variability within local authorities: UK 2015-2019. Table S2. Mixed-effects linear regression model for selected components and their selected 2-way interaction terms vs. food desert risk (z-scored) at the LSOA/DZ levels for each nation, with random effect added for intercept to account for variability within local authorities, adjusting for socio-demographic classification at the LSOA/DZ levels: England and Wales 2015-2019.

Supplementary Material 2.

## Data Availability

Most data used in this study are publicly available through the Consumer Data Research Centre and the Geographic Data Service ([https://www.data.hasp.ac.uk] (https://www.data.hasp.ac.uk) and [https://data.geods.ac.uk] (https://data.geods.ac.uk)). Derived datasets, statistical code, figures, and documentation supporting the findings are available at the project GitHub repository: (https://github.com/baydounm/UK_PAPER20_FOODDESERTIMD). Some safeguarded data accessed through approved research applications cannot be redistributed but can be accessed directly from the data providers subject to their governance procedures.

## Abbreviations

CI: Confidence interval
CDRC: Consumer Data Research Centre
DZ: Data Zone
EFDI: E-Food Desert Index
IMD: Index of Multiple Deprivation
LA: Local Authority
LASSO: Least absolute shrinkage and selection operator
LSOA: Lower Layer Super Output Area
OLS: Ordinary least squares
RRR: Relative risk ratio
SD: Standard deviation
UK: United Kingdom
