# Supplementary material for: Mapping food access: how neighborhood deprivation shapes healthy food availability in the United Kingdom
Source: BMC Public Health. 2026 Feb 23;26:1048. doi: 10.1186/s12889-026-26664-2 (PMC13037262; doi:10.1186/s12889-026-26664-2)
Supplement: Supplementary file 1 — Supplementary Material 1. Table S1. Mixed-effects linear regression model for selected components and their selected 2-way interaction terms vs. food desert risk (z-scored) at the LSOA/DZ levels for each nation, with random effect added for intercept to account for variability within local authorities: UK 2015-2019. Table S2. Mixed-effects linear regression model for selected components and their selected 2-way interaction terms vs. food desert risk (z-scored) at the LSOA/DZ levels for each nation, with random effect added for intercept to account for variability within local authorities, adjusting for socio-demographic classification at the LSOA/DZ levels: England and Wales 2015-2019. [file 12889_2026_26664_MOESM1_ESM.pdf]

APPENDICES

APPENDIX 1: LASSO Regression Analysis

The dataset used for the LASSO regression models was obtained through the combination of several datasets from the UK CDRC data resource. This data resource includes comprehensive indices of deprivation and demographic variables for England, Scotland, and Wales. The variables selected for each country were based on their relevance to socio-economic deprivation and potential impact on food accessibility. Standardization of variables was performed using the *egen* command in Stata to create z-scores, facilitating comparability across different scales. The dataset for each country was split into training and validation subsets using the *splitsample* command with a random seed set to 1234 to ensure reproducibility.

LASSO regression was conducted using the lasso linear command in Stata, with the regularization parameter (λ) tuned through cross-validation. The *cvplot* command was utilized to visualize cross-validation results, and the *lassoknots* command provided information on the number of non-zero coefficients, out-of-sample R-squared values, and BIC for different λ values. The model corresponding to the minimum BIC was selected as the final model. Adaptive LASSO was also performed using the selection option within the lasso linear Stata command. To examine complex relationships among predictors while maintaining model parsimony, we implemented a hierarchical least absolute shrinkage and selection operator (LASSO) procedure that incorporates interaction terms under the strong heredity principle, in addition to the model that did not include any interactions, but only the main effects of the components of IMD within each nation (England, Scottland and Wales).

1. LASSO Regression

The Least Absolute Shrinkage and Selection Operator (LASSO) estimates regression coefficients by minimizing the following penalized loss function:

$$\beta^{\widehat{LASSO}} = \arg min_{\beta} \left\{ \frac{1}{2n} \sum_{i=1}^{n} (y_i - x_i^T \beta)^2 + \lambda \sum_{j=1}^{p} |\beta_j| \right\}$$

y_i: outcome variable, in this case z-scored food desert risk value.

x_i:predictor vector for i-th observation, in this case the components of the IMD, z-scored.

$\lambda \geq 0$: tuning parameter controlling penalty strength.

The L1-penalty term $\sum_{j=1}^{p} |\beta_j|$ encourages sparsity by shrinking some coefficients exactly to zero.

***Reference**: [1-4]*

2. Cross-Validated LASSO (cvLASSO)

cvLASSO selects the tuning parameter λ through K-fold cross-validation, minimizing the mean cross-validated prediction error:

$$\hat{\lambda}_{cv} = \arg min_{\lambda} \left(\frac{1}{K} \sum_{k=1}^{K} CV_k(\lambda) \right)$$

This is often applied to 10-fold cross-validation and the model uses λcv to estimate β.

This method balances model fit and generalizability.

***Reference**: [1-4]*

3. Minimum BIC LASSO (minBIC LASSO)

The minBIC LASSO selects λ by minimizing the Bayesian Information Criterion (BIC), calculated for each candidate λ:

$$BIC(\lambda) = nlog(\hat{\sigma}_{\lambda}^2) + \log(\mathrm{n}) \,.\, \mathrm{df}_{\lambda}$$

$\hat{\sigma}_{\lambda}^2$: residual variance under LASSO with penalty λ.

df_{λ}: degrees of freedom, typically the number of non-zero coefficients.

n: sample size.

The BIC criterion favors models that fit well while penalizing model complexity. minBIC tends to select **sparser** models than cvLASSO.

***Reference**: [1-4]*

4. Adaptive LASSO

The adaptive LASSO improves upon standard LASSO by assigning adaptive weights wjw_jwj to each coefficient in the penalty term:

$$\hat{\beta}^{ALASSO} = \arg min_{\beta} \left\{ \frac{1}{2n} \sum_{i=1}^{n} (y_i - x_i^T \beta)^2 + \lambda \sum\nolimits_{j=1}^{p} w_j |\beta_j| \right\}$$

$wj = 1/|\hat{\beta}_i^{init}|^{\gamma}$, where $\hat{\beta}_i^{init}$ is a root-n consistent estimator (e.g., OLS, or ridge).

γ>0: tuning parameter (typically set to 1).

Adaptive LASSO enjoys the oracle property, meaning it can identify the true model asymptotically.

Reference: [5, 6]

APPENDIX II: *LASSO Regression Findings with main effects*

ENGLAND

	cvENGLAND	minBICENGLAND	adaptiveENGLAND
zemploymentscorerate	x	x	x
zincomescorerate	x	x	x
zcrimescore	x	x	x
zbarrierstohousingandservscore	x	x	x
zeducationskillsandtrainingscore	x	x	x
zhealthdeprivationanddisabsc	x	x	x
zlivingenvironmentscore	x	x	x
_cons	x	x	x

Legend:
b - base level
e - empty cell
o - omitted
x - estimated

Postselection coefficients

Name	sample_L~D	MSE	R-squared	Obs
cvENGLAND				
	1	.4744777	0.4198	16,422
	2	.4817145	0.4231	16,422
minBICENGLAND				
	1	.4744777	0.4198	16,422
	2	.4817145	0.4231	16,422
adaptiveENGLAND				
	1	.4744777	0.4198	16,422
	2	.4817145	0.4231	16,422

SCOTTLAND

	cvSCOTLAND	minBICSCOTLAND	adaptiveSCOTLAND
zsimd2020v2_income_domain_rank	x	x	
zsimd2020_access_domain_rank	x	x	
zsimd2020_employment_domain_rank	x	x	x
zsimd2020_crime_domain_rank	x	x	
zsimd2020_housing_domain_rank	x	x	x
zsimd2020_education_domain_rank	x	x	x
_cons	x	x	x

Legend:
b - base level
e - empty cell
o - omitted
x - estimated

Postselection coefficients

Name	samp~TLAND	MSE	R-squared	Obs
cvSCOTLAND				
	1	.9659442	0.3761	3,488
	2	.9942468	0.3676	3,488
minBICSCOTLAND				
	1	.9659442	0.3761	3,488
	2	.9942468	0.3676	3,488
adaptiveSCOTLAND				
	1	.0559045	0.9442	3,488
	2	.0568349	0.9431	3,488

WALES

	cvWALES	minBICWALES	adaptiveWALES
zAccessServices_Score2019	x	x	
zEmployment_Score2019	x	x	x
zHealth_Score2019	x	x	x
zCommunitySafety_Score2019	x	x	
zPhysicalEnvironment_Score2019	x	x	
zIncome_Score2019	x	x	
zHousing_Score2019	x	x	
zEducation_Score2019			x
_cons	x	x	x

Legend:
- b - base level
- e - empty cell
- o - omitted
- x - estimated

Postselection coefficients

Name	sample_L~S	MSE	R-squared	Obs
cvWALES				
	1	.5824592	0.6332	955
	2	.6391186	0.6136	954
minBICWALES				
	1	.5824592	0.6332	955
	2	.6391186	0.6136	954
adaptiveWALES				
	1	.0680789	0.9349	955
	2	.0756418	0.9205	954

APPENDIX III: Index of Multiple Deprivation and components

General Pattern (All Nations)

- IMD Scores:
  Higher score = More deprivation
- IMD Ranks:
  Rank 1 = Most deprived area
  Higher rank (e.g. 32,844) = Less deprived area

England IMD Components

The English IMD comprises seven weighted domains, with the following scores and ranks:

Domain	Score Direction	Rank Direction
Income	Higher score = More income deprivation	Rank 1 = Most deprived
Employment	Higher score = More employment deprivation	Rank 1 = Most deprived
Education, Skills and Training	Higher score = More deprivation in education/training	Rank 1 = Most deprived
Health Deprivation and Disability	Higher score = Poorer health outcomes, more deprivation	Rank 1 = Most deprived
Crime	Higher score = More crime-related deprivation	Rank 1 = Most deprived

Domain	Score Direction	Rank Direction
Barriers to Housing and Services	Higher score = Greater geographic/financial barriers	Rank 1 = Most deprived
Living Environment	Higher score = Worse indoor/outdoor conditions	Rank 1 = Most deprived

Overall IMD Score and Rank:
Weighted combination of domain scores

- Higher overall score = More deprived
- Rank 1 = Most deprived LSOA out of ~32,844 in England

Scotland SIMD Components

Scottish Index of Multiple Deprivation (SIMD) uses data zones and similar domain structure:

Domain	Score Direction	Rank Direction
Income	Higher score = More deprivation	Rank 1 = Most deprived
Employment	Higher score = More deprivation	Rank 1 = Most deprived
Education	Higher score = More deprivation	Rank 1 = Most deprived
Health	Higher score = More deprivation	Rank 1 = Most deprived
Access to Services	Higher score = Poorer access (more deprived)	Rank 1 = Most deprived
Crime	Higher score = Higher crime rate (more deprived)	Rank 1 = Most deprived
Housing	Higher score = Poorer housing conditions	Rank 1 = Most deprived

SIMD Score and Rank:

- Higher SIMD score = More deprived
- Rank 1 = Most deprived of ~6,976 data zones

Wales WIMD Components

Welsh Index of Multiple Deprivation (WIMD) includes:

Domain	Score Direction	Rank Direction
Income	Higher score = More income-related deprivation	Rank 1 = Most deprived
Employment	Higher score = More employment deprivation	Rank 1 = Most deprived

Domain	Score Direction	Rank Direction
Health	Higher score = Poorer health outcomes	Rank 1 = Most deprived
Education	Higher score = More deprivation in skills/attainment	Rank 1 = Most deprived
Access to Services	Higher score = Poorer access to services	Rank 1 = Most deprived
Community Safety	Higher score = Less safe (more deprivation)	Rank 1 = Most deprived
Physical Environment	Higher score = More environmental deprivation	Rank 1 = Most deprived
Housing	Higher score = Greater housing deprivation	Rank 1 = Most deprived

WIMD Score and Rank:

- Higher WIMD score = More deprived
- Rank 1 = Most deprived of ~1,909 LSOAs in Wales

Summary of Directionality:

Type	Higher Score Means	Lower Rank Means
Score	More deprivation	Not applicable
Rank	Not applicable	More deprivation

Table S1. Mixed-effects linear regression model for selected components and their selected 2-way interaction terms vs. food desert risk (z-scored) at the LSOA/DZ levels for each nation, with random effect added for intercept to account for variability within local authorities: UK 2015-2019

	**Y=Food desert risk, z-scored**		
	**England**	**Scotland**	**Wales**
	β±SE	β±SE	β±SE
	**Score**	**Rank**	**Score**
	**↑ score ↑ deprivation**	**↑ rank ↓ deprivation**	**↑ score ↑ deprivation**
	**N=32,844**	**N=6,976**	**N=1,909**
**X=Component of multiple deprivation and their 2-way interactions, z-scored[a]**			
Main effects			
Income score/rank	+0.142±0.016***	-0.423±0.043***	+0.419±0.068***
Employment score/rank	+0.422±0.017***	-0.277±0.038***	+0.509±0.056***
Education score/rank	+0.0003±0.0084	-0.031±0.023	…
Health score/rank	-0.084±0.010***	…	-0.371±0.057***
Housing score/rank	… [b]	-0.013±0.019	-0.330±0.031***
Crime score/rank	-0.167±0.005***	+0.171±0.014***	…
Access to services score/rank	…[b]	-0.402±0.011***	+0.417±0.025***
Housing and access to service score/rank	+0.240±0.005***	…	…
Living Environment	+0.087±0.005***	…	-0.003±0.001*
Two-way interaction terms			
Health×Income	+0.210±0.019***	…	…
Housing/service×Income	-0.116±0.011***	…	…
Education×Health	-0.034±0.013**	…	+0.060±0.021**
Employment×Income	-0.109±0.010***	+0.138±0.028***	-0.239±0.039***
Crime×Income	+0.121±0.015***	+0.111±0.048*	…
Employment×Housing/service	+0.125±0.012***	…	…
Education×Employment	+0.119±0.015***	+0.200±0.048***	…
Crime×Living environment	-0.095±0.005***	…	+0.0020±0.0008**
Health×Housing/service	-0.086±0.007***	…	
Crime×Employment	-0.079±0.016***	-0.182±0.043***	…
Housing/service×Living environment	+0.089±0.003***	…	…
Employment×Health	-0.031±0.016*	…	+0.159±0.041
Income×Living environment	-0.035±0.013**	…	…
Employment×Living environment	+0.049±0.012***	…	…
Education×Living environment	+0.032±0.007***	…	…
Eduction×Income	-0.074±0.014***	-0.187±0.049***	
Crime× Housing/service	-0.0004±0.0043	…	…
Crime×Health	+0.025±0.008**	…	+0.100±0.018***
Health×Living environment	-0.064±0.007***	…	…
Education×Housing/services	+0.002±0.007	…	…
Crime×Education	-0.031±0.008***	+0.097±0.026***	…
Service×Income	…	-0.042±0.043	…
Service×Employment	…	-0.043±0.037	…
Service×Housing	…	+0.073±0.015***	…
Crime×Housing	…	-0.118±0.018***	-0.072±0.018***
Service×Income	…	-0.041±0.043	…
Service×Education	…	*-0.041±0.024*	…
Housing×Income	…	+0.056±0.027*	+0.037±0.019

Service×Health	…	…	-0.150±0.033
Service×Crime	…	-0.164±0.014***	-0.088±0.028***
Housing×Living environment	…	…	-0.0024±0.0006***
Housing×Education	…	-0.099±0.025***	…
Intercept	-0.007±0.027	+0.281±0.139*	+0.493±0.100***
Number of clusters	317	32	22
var(_cons)	0.229 (0.019)	0.607 (0.154)	0.203 (0.604)
var(Residual)	0.267 (0.002)	0.621 (0.011)	0.411 (0.013)
ICC	0.46	0.49	0.33

Abbreviations: CI=Confidence Interval; DZ=Data Zone; ICC=Intra-cluster correlation; IMD=Index of Multiple Deprivation; LASSO=Least Absolute Shrinkage Selection Operator; LSOA=Lower Statistical Output Area; SE=Standard Error; UK=United Kingdom.

[a] Component of IMD scores were used for England and Wales, whereas ranks of these component scores were used for Scotland. In all cases, predictors were z-score standardized. The crime score for England was Ln transformed before z-score standardization after adding 10 as a constant to ensure there are no negative values. Model selected using a hierarchical adaptive LASSO machine learning technique with 2-way interaction terms included for all available IMD components.

[b] Access to service and housing scores were combined for England.

[c] A sensitivity analysis was implemented by using a series of linear mixed-effect regression models with a random effect added to the intercept to account for variability within local authorities.

*P<0.05; **P<0.010; ***P<0.001

Sources: [7-9]

Table S2. Mixed-effects linear regression model for selected components and their selected 2-way interaction terms vs. food desert risk (z-scored) at the LSOA/DZ levels for each nation, with random effect added for intercept to account for variability within local authorities, adjusting for socio-demographic classification at the LSOA/DZ levels: England and Wales 2015-2019

	**Y=Food desert risk, z-scored**	
	**England**	**Wales**
	β±SE	β±SE
	**Score**	**Score**
	**↑ score ↑ deprivation**	**↑ score ↑ deprivation**
	**N=31,810**	**N=1,837**
**X=Component of multiple deprivation and their 2-way interactions, z-scored**[a]		
Main effects		
Income score/rank	+0.263±0.017***	+0.402±0.069***
Employment score/rank	+0.347±0.017***	+0.507±0.057***
Education score/rank	+0.021±0.008*	…
Health score/rank	-0.068±0.010***	-0.303±0.057***
Housing score/rank	… [b]	+0.159±0.027***
Crime score/rank	-0.152±0.005***	…
Access to services score/rank	…[b]	+0.414±0.026***
Housing and access to service score/rank	+0.235±0.004***	…
Living Environment	+0.119±0.005***	-0.003±0.001*
Two-way interaction terms		
Health×Income	+0.185±0.019***	…
Housing/service×Income	-0.137±0.011***	…
Education×Health	-0.023±0.013	+0.049±0.021*
Employment×Income	-0.098±0.010***	-0.203±0.038*
Crime×Income	+0.092±0.015***	…
Employment×Housing/service	+0.117±0.012***	…
Education×Employment	+0.119±0.015***	…
Crime×Living environment	-0.089±0.0041***	+0.0017±0.0008*
Health×Housing/service	-0.067±0.007***	…
Crime×Employment	-0.056±0.016***	…
Housing/service×Living environment	+0.0757±0.003***	…
Employment×Health	-0.023±0.016	+0.122±0.041**
Income×Living environment	-0.039±0.013**	…
Employment×Living environment	+0.039±0.012**	…
Education×Living environment	+0.033±0.007***	…
Education×Income	-0.092±0.014***	…
Crime× Housing/service	-0.001±0.004	…
Crime×Health	+0.023±0.008**	+0.109±0.020***
Health×Living environment	-0.054±0.007***	…
Education×Housing/services	+0.013±0.007*	…
Crime×Education	-0.029±0.008**	…
Service×Income	…	…
Service×Employment	…	…
Service×Housing	…	…
Crime×Housing	…	-0.045±0.019*
Service×Income	…	…
Service×Education	…	…

Housing×Income	…	+0.003±0.020
Service×Health	…	-0.117±0.034***
Service×Crime	…	-0.112±0.034***
Housing×Living environment	…	-0.0021±0.0007***
Housing×Education	…	…
Socio-demographic categories		
1. Retired professionals (referent)	—	—
2. Suburbanites and Peri-Urbanites	-0.036±0.010***	+0.043±0.044
3. Multicultural and educated urbanites	-0.519±0.021***	-0.659±0.140***
4. low-skilled migrant and student communities	-0.474±0.017***	-0.410±0.147**
5. Ethnically diverse sub-urban professionals	-0.316±0.013***	-0.397±0.104***
6. Baseline UK	-0.416±0.014***	-0.522±0.076***
7. Semi- and Un-Skilled Workforce	-0.232±0.014***	-0.135±0.060*
8. Legacy Communities	-0.025±0.034	-0.084±0.164
Intercept	+0.243±0.026***	+0.577±0.102***

Abbreviations: CI=Confidence Interval; DZ=Data Zone; IMD=Index of Multiple Deprivation; LASSO=Least Absolute Shrinkage Selection Operator; LSOA=Lower Statistical Output Area; SE=Standard Error; UK=United Kingdom.

[a] Component of IMD scores were used for England and Wales, whereas ranks of these component scores were used for Scotland. In all cases, predictors were z-score standardized. The crime score for England was Ln transformed before z-score standardization after adding 10 as a constant to ensure there are no negative values. Model selected using a hierarchical adaptive LASSO machine learning technique with 2-way interaction terms included for all available IMD components.

[b] Access to service and housing scores were combined for England.

[c] A sensitivity analysis was implemented by using a series of linear mixed-effect regression models with a random effect added to the intercept to account for variability within local authorities. This sensitivity analysis builds on the previous one by adding an LSOA-level socio-demographic classification variable.

*P<0.05; **P<0.010; ***P<0.001

Sources: [7-9] and https://data.geods.ac.uk/dataset/lsoac

References

1. Tibshirani R: **The lasso method for variable selection in the Cox model**. *Stat Med* 1997, **16**(4):385–395.
2. Bien J, Taylor J, Tibshirani R: **A Lasso for Hierarchical Interactions**. *Ann Stat* 2013, **41**(3):1111–1141.
3. Friedman J, Hastie T, Tibshirani R: **Sparse inverse covariance estimation with the graphical lasso**. *Biostatistics* 2008, **9**(3):432–441.
4. Lockhart R, Taylor J, Tibshirani RJ, Tibshirani R: **A Significance Test for the Lasso**. *Ann Stat* 2014, **42**(2):413–468.
5. Bahamyirou A, Schnitzer ME, Kennedy EH, Blais L, Yang Y: **Doubly robust adaptive LASSO for effect modifier discovery**. *Int J Biostat* 2022, **18**(2):307–327.
6. Foster JC, Taylor JM, Nan B: **Variable selection in monotone single-index models via the adaptive LASSO**. *Stat Med* 2013, **32**(22):3944–3954.
7. **2011 Census Geography boundaries (Lower Layer Super Output Areas and Data Zones)** [https://statistics.ukdataservice.ac.uk/dataset/2011-census-geography-boundaries-lower-layer-super-output-areas-and-data-zones]
8. Newing A. VF: **E-food Desert Index (EFDI) Technical report and user guide. URL: https://data.hasp.ac.uk/browser/dataset/5347/0**. In.; 2020.
9. **Index of Multiple Deprivation (IMD)** [https://data.geods.ac.uk/dataset/index-of-multiple-deprivation-imd]
